# Associations of epigenetic aging and COVID- 19: A 3-year longitudinal study

**DOI:** 10.1007/s11357-025-01635-4

**Published:** 2025-04-10

**Authors:** Gabor Farkas, Zahira El Mahdaouy, Gergely Babszky, Matyas Jokai, Ferenc Torma, Yaodong Gu, Ricardo Pinho, Ildiko Miklossy, Juozas Gordevicius, András Benczúr, Csaba Kerepesi, Zsolt Radak

**Affiliations:** 1 Research Institute of Sport Science, Hungarian University of Sport Science, Budapest, Hungary; 2 Institute for Computer Science and Control (SZTAKI), Hungarian Research Network (HUN-REN), Budapest, Hungary; 3 Faculty of Sport Science, Ningbo University, Ningbo, 315211 China; 4 Laboratório de Bioquímica do Exercício em Saúde, Programa de Pós-Graduação em Ciências da Saúde, Escola de Medicina e Ciências da Vida, Pontifícia Universidade Católica Do Paraná, Curitiba, PR Brazil; 5 Department of Bioengineering, Sapientia Hungarian University of Transylvania, Piata 26 Libertatii, 530104 Miercurea Ciuc, Romania; 6 Faculty of Sport Sciences, Waseda University, Tokorozawa, Japan; 7 Institute of Sport Sciences and Physical Education, Faculty of Sciences, University of Pécs, 247624 Pécs, Hungary; 8 Epigenetic Clock Development Foundation, Torrance, CA USA

**Keywords:** Epigenetic aging, COVID- 19, Epigenetic clocks, Longitudinal aging, H1 FNT

## Abstract

Aging and COVID- 19 are known to influence DNA methylation, potentially affecting the rate of aging and the risk of disease. The physiological functions of 54 volunteers—including maximal oxygen uptake (VO₂ max), grip strength, and vertical jump—were assessed just before the COVID- 19 pandemic and again 3 years later. Of these volunteers, 27 had contracted COVID- 19. Eight epigenetic clocks were used to assess the rate of aging during the 3-year period: DNAmAge showed accelerated aging, and five clocks showed slowed aging (DNAmAgeSkinBlood, DNAmAgeHannum, DNAmFitAge, PhenoAge, and DNAmTL). When we considered only females, we observed a stronger effect in the increase of DNAmAge acceleration, while we observed slowed aging in the case of SkinBloodClock, and DNAmTL. The methylation of the promoter region of the H1 FNT genes, which encodes testis-specific histone H1 family member N (H1fnt) and plays a crucial role in spermatogenesis decreased the most significantly. In contrast, the promoter of CSTL1, which encodes Cystatin-like 1, showed the most significant increase. We found that having COVID- 19 during the 3-year study period significantly increased the progress of aging assessed by DNAmGrimAge, DNAmGrimAge2, and DNAmFitAge (*p* = 0.024, 0.047, 0.032, respectively, after we adjusted the analysis for baseline variables). The data suggest that COVID- 19 may have a mild long-term effect on epigenetic aging.

**Supplementary Information:**

The online version contains supplementary material available at 10.1007/s11357-025-01635-4.

## Introduction

The infection caused by severe acute respiratory syndrome coronavirus 2 (SARS-CoV- 2) and the associated coronavirus disease 2019 (COVID- 19) pandemic drastically changed the lifestyle of billions of people, significantly impacting health, mental status, working conditions, and levels of physical activity. Each of these changes can affect the epigenetics of individuals, thereby altering gene expression, susceptibility to various diseases, and the rate of aging. Indeed, DNA methylation, which is the most well-studied epigenetic modification, is a reversible and environment-dependent process. The DNA methylation pattern serves as cellular memory, ensuring that cells maintain their specific functions and characteristics. A strong link has been established between DNA methylation and disease susceptibility, mortality, and the progression of aging [1–3].

COVID- 19 infection has serious acute and long-term consequences, involving cellular memory. It has been shown that the methylation of specific CpG sites can distinguish COVID- 19-infected patients from healthy controls [4]. Balnis et al. reported that immediately after COVID- 19 infection, 72% of patient’s blood samples showed hypomethylation, while 28% showed hypermethylation [5]. The study also indicated that hypomethylation occurred in the promoter regions of certain genes, leading to immunosuppression.

Pang et al. studied the DNA methylation states of 21 subjects before and after COVID- 19 diagnosis [6]. The epigenetic clock estimates of PhenoAge and GrimAge significantly increased in subjects over 50 years old by an average of 2.1 and 0.84 years, respectively, following infection, even though the time between samplings was just 8.4 weeks. On the other hand, the principal component-based PhenoAge significantly decreased in people under 50 following infection by an average of 2.06 years [6]. It was suggested that this discrepancy was due to changes in immune cell composition.

A significant number of studies have examined the acute and long-term consequences of COVID- 19 on health. Measuring DNA methylation-based aging in healthy and COVID- 19-infected subjects revealed an increase in the acceleration of epigenetic aging and telomere attrition with COVID- 19 infection. Accelerated epigenetic aging is associated with a higher risk of SARS-CoV- 2 infection and the development of severe COVID- 19 [7–9].

Despite the accumulation of information on COVID- 19’s effects on DNA methylation and DNA methylation-based aging, many pieces of the puzzle are still missing in fully understanding how COVID- 19 impacts DNA methylation and aging clocks. In this study, we collected pre- and post-COVID- 19 samples over a time frame of 3 years and examined the associations of epigenetic age acceleration and COVID- 19.

## Results

### Baseline characteristics of our cohort

We assessed the physical fitness and blood epigenetics of 54 study participants (35 females and 19 males), all middle-aged or older (41–83 years), except for one younger participant (25 years old). Highly educated people were overrepresented in our cohort with two-thirds of them having at least BSc degree (see Table 1). Measurements were taken at two time points: just before the onset of the COVID- 19 pandemic (between 29/10/2019 and 21/11/2019) and approximately 3 years later, near the end of the global pandemic (between 17/10/2022 and 19/01/2023). Among the 54 participants, 27 reported having had COVID- 19 at some point during the study period. For simplicity, we refer to this group as “infected,” though no formal confirmation of SARS-CoV- 2 infection was required.

**Table 1:** Baseline characteristics of the first measurement (in November/December 2019) of the 54 study participants. Some participants had COVID- 19 disease (*Infected*) or did not have COVID- 19 (*Noninfected*) within a 3-year study period

**Numerical variable**	***n***	**Mean**	**Std**	***p*-value**
Age (infected)	27	56.224	13.26	
Age (noninfected)	27	61.336	11.577	0.137
VO2 max BEFORE: (infected)	21	34.025	9.147	
VO2 max BEFORE: (noninfected)	26	33.154	6.555	0.716
Max Jump BEFORE (infected)	23	25.652	7.033	
Max Jump BEFORE (noninfected)	24	26.179	7.735	0.808
Max Grip BEFORE (infected)	25	39.472	12.549	
Max Grip BEFORE (noninfected)	27	35.715	14.339	0.319
Kognitiv BEFORE (infected)	26	6.231	1.366	
Kognitiv BEFORE (noninfected)	27	6.074	1.299	0.671
BMI (infected)	26	28.817	4.84	
BMI (noninfected)	27	26.507	3.907	0.063
Body mass before (infected)	26	83.773	21.043	
Body mass BEFORE (noninfected)	27	72.511	13.168	0.025
Body height BEFORE (infected)	26	168.6	7.736	
Body height BEFORE (noninfected)	27	165.17	9.952	0.167
Years of regular training: (infected)	26	13.115	13.56	
Years of regular training: (noninfected)	27	12.148	14.567	0.803
Hours per week: (infected)	14	1.786	2.045	
Hours per week: (noninfected)	10	3.2	3.36	0.257
**Categorical variable**	**Category**	**Infected count**	**Noninfected count**	***p*-value**
Gender	Female	16	19	0.5694
Gender	Male	11	8	0.5694
Education degree	University	10	6	0.3718
Education degree	High School	8	8	1
Education degree	Elementary	2	0	0.4906
Education degree	PhD	3	2	1
Education degree	BSc	4	11	0.0664
Nutritional habits	Diabetes diet	0	1	1
Nutritional habits	Vegetarian	1	2	1
Nutritional habits	Sugar-free diet	1	0	0.4906
Nutritional habits	Normal	24	24	1
Diseases	Hypothyroidism	1	0	0.4828
Diseases	Reflux	0	1	1
Diseases	Psychiatric disorder	0	1	1
Diseases	No reported disease	18	16	0.4351
Diseases	Polyarthritis	0	1	1
Diseases	Autoimmune	1	1	1
Diseases	Diabetes	1	0	0.4828
Diseases	Acute infection	1	0	0.4828
Diseases	High blood pressure	4	8	0.3356
Diseases	Fever	1	0	0.4828
Diseases	Epilepsy	1	0	0.4828
Diseases	Asthma	0	2	0.4918
Vaccination	Not vaccinated	3	3	1
Vaccination	Moderna	3	3	1
Vaccination	AstraZeneca	4	4	1
Vaccination	Pfizer	16	21	0.8197
Vaccination	Sinopharm	3	3	1
Vaccination	Sputnik V	6	8	1
Vaccination dose number	1	6	11	0.2683
Vaccination dose number	2	13	17	0.6160
Vaccination dose number	3	10	5	0.1386
Vaccination dose number	4	1	0	0.4762

### Increased physical fitness of middle-aged and older individuals during the COVID- 19 pandemic except for the SARS-CoV- 2 infected ones

**Fig. 1:**
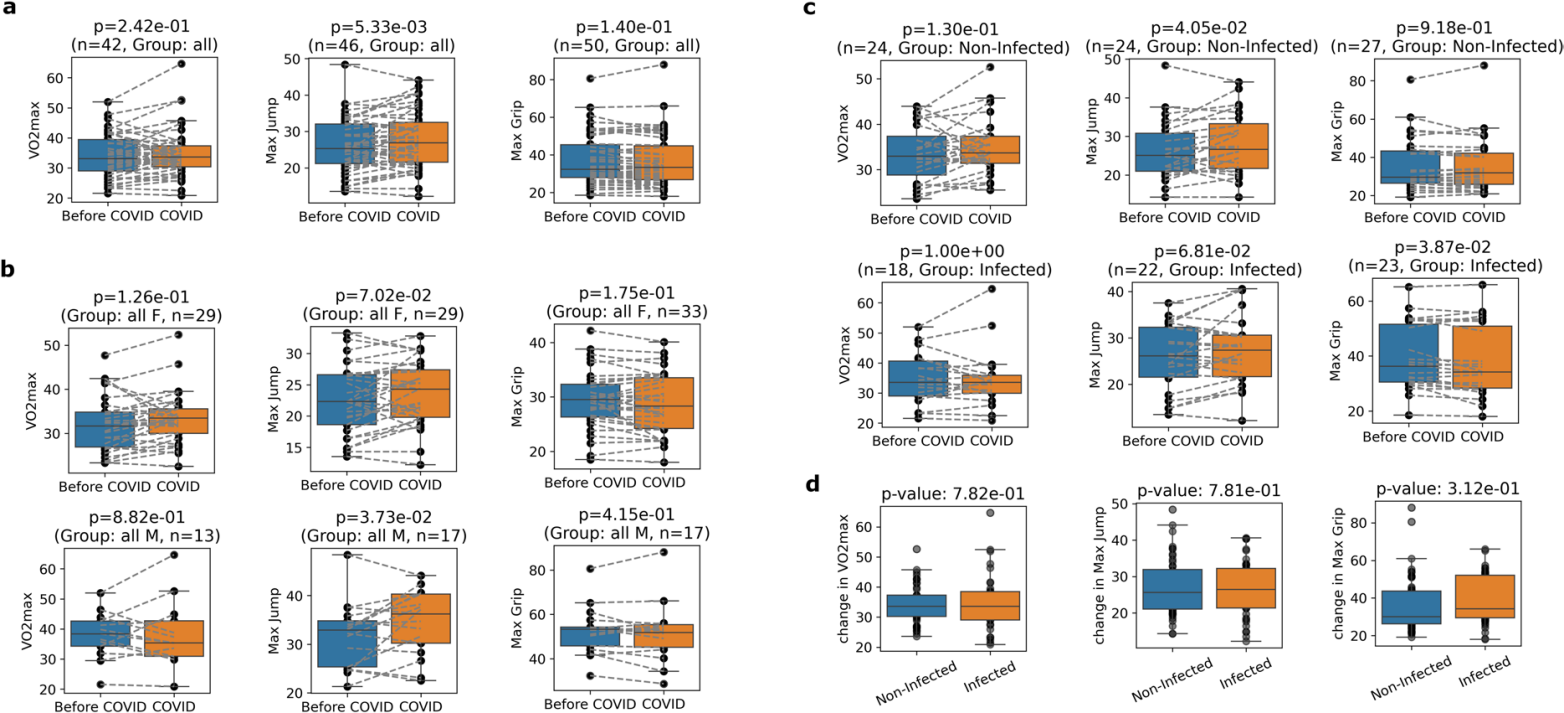
Increased physical fitness of middle-aged and older individuals during the COVID- 19 pandemic except for SARS-CoV- 2 infected ones. **a** Maximal relative oxygen uptake (*VO2 max*), maximum vertical jump (*Max Jump*), and maximum handgrip force (*Max Grip*) measured just before the COVID- 19 pandemic (*Before COVID*) and 3 years later during the COVID- 19 pandemic (*COVID*). All participants are included (*n* = 54) regardless of the infection status. **b** The same analysis with the separation of genders (*females* and *males)*. **c** The same analysis with the separation by SARS-CoV- 2 infection status (*infected* and *non-infected*). **d** Comparison of the 3-year-change of fitness for infected and non-infected groups

As we examined almost exclusively middle-aged and older individuals, we did not expect improved fitness with age. Instead, we observed a significant increase in the maximum vertical jump during the examined 3-year period of the COVID- 19 pandemic and no significant change in maximal relative oxygen uptake (VO2 max) and maximum handgrip force (Fig. 1a). When we separated the analysis by gender, the max jump increase remained significant only for males (Fig. 1b).

However, maximal jump performance did not change significantly in individuals who had COVID- 19 during the 3-year study period. In contrast, maximal grip strength showed a significant decrease (Fig. 1c). Furthermore, we compared the 3-year-change of fitness of infected and non-infected groups and found no significant difference (Fig. 1d). In summary, our data show that physical fitness of middle aged and older people increased during the COVID- 19 pandemic except for the SARS-CoV- 2 infected ones.

### Associations of DNA methylation-based aging and COVID- 19 during the 3-year study period

We measured DNA methylation levels at approximately 850,000 CpG sites in all 54 study participants at two time points: just before the COVID- 19 pandemic and again 3 years later. The principal component analysis (PCA) of methylomes showed a clear separation by gender but no sharp separation by time point, indicating no visible batch effect (see Supplementary Fig. S1).

We examined the 3-year changes in methylation levels for each individual exploring the natural aging process in human blood. Among the top 20 differently methylated gene promoters, 13 showed hypermethylation, and 7 showed hypomethylation (Supplementary Fig. S2; Supplementary Table S1). The most significant decrease was shown by the promoter of the H1 FNT gene (mean promoter methylation dropped from 0.883 to 0.821, FDR adjusted *p*-value = 8.35E − 33), while the most significant increase was shown by the promoter of CSTL1 (mean promoter methylation elevated from 0.772 to 0.839, FDR adjusted *p*-value = 2.65E − 31). The majority of the top 20 genes remained in the top 20 when considering only participants who were (i) infected (*n* = 27, Supplementary Fig. S3; Supplementary Table S2) or (ii) non-infected (*n* = 27, Supplementary Fig. S4; Supplementary Table S3) by SARS-CoV- 2 during the 3-year study period, with 15 and 11 genes, respectively, remaining in the top 20. We also examined whether SARS-CoV- 2 infection at any point during the study period affected the 3-year changes of promoter methylation, but we did not find any significant differences (Supplementary Table S4).

**Fig. 2:**
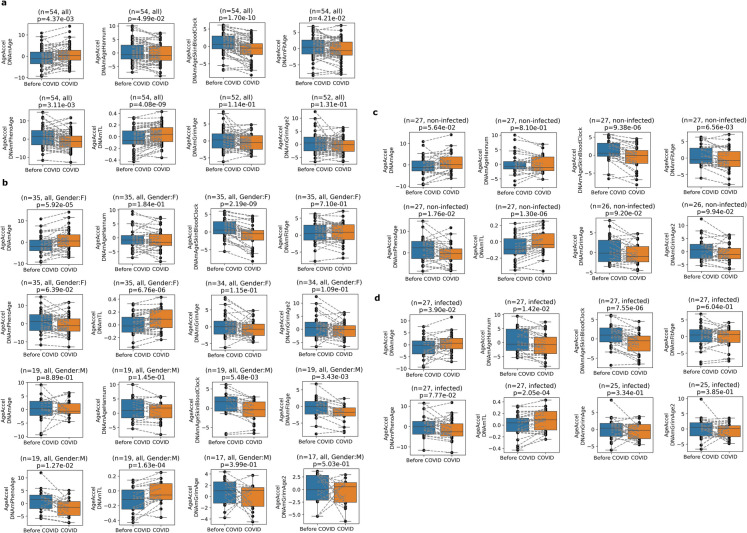
Mixed changes of epigenetic aging in middle-aged and older individuals during the COVID- 19 pandemic. **a** Age acceleration of eight aging clocks (DNAmAge, DNAmAgeHannum, DNAmAgeSkinBloodClock, DNAmFitAge, PhenoAge, DNAmTL, DNAmGrimAge, and DNAmGrimAge2) measured just before the COVID- 19 pandemic (*Before COVID*) and 3 years later during the COVID- 19 pandemic (*COVID*). All participants are included regardless of the infection status. **b** The same analysis with the separation of genders (*females* and *males*). **c** The same analysis for subjects who were not infected by SARS-CoV- 2 during the examined 3-year period (*non-infected*)*.* **d** The same analysis for subjects who were infected by SARS-CoV- 2 during the examined 3-year period (*infected)*

We also examined seven epigenetic aging clocks (i.e., age predictor models based on methylation levels of certain CpG sites) and the DNA methylation-based predictor of telomere length (DNAmTL). As expected, the prediction performance of the seven epigenetic aging clocks showed a high positive correlation with age (Pearson *r* = 0.9–0.96), while the DNAmTL showed a negative correlation with *r* = − 0.74 (Supplementary Fig. S5). Then, we evaluated the 3-year changes in age acceleration by using the eight clocks. One clock, the multi-tissue clock (DNAmAge), showed accelerated aging, and five clocks showed slowed aging (DNAmAgeSkinBlood, DNAmAgeHannum, DNAmFitAge, PhenoAge, and DNAmTL), while the remaining two clocks (DNAmGrimAge and DNAmGrimAge2) did not show a significant change in age acceleration (Fig. 2a). We note that since telomere length decreases with age, an increase in age acceleration of DNAmTL is interpreted as slowed aging. When we considered only females, we observed a stronger effect in the increase of DNAmAge acceleration while we observed slowed aging in the case of SkinBloodClock and DNAmTL (Fig. 2b). Interestingly, when we considered only males, we did not observe a significant change in DNAmAge age acceleration; however, four clocks (DNAmAgeSkinBlood, DNAmFitAge, PhenoAge, and DNAmTL) still showed slowed aging (Fig. 2b). Individuals who were not infected by SARS-CoV- 2 during the 3-year study period exhibited slowed aging by DNAmAgeSkinBlood, DNAmFitAge, PhenoAge, and DNAmTL (Fig. 2c; Supplementary Table S2). Individuals who were infected by SARS-CoV- 2 during the 3-year study period showed a significant increase in DNAmAge and slowed aging by DNAmAgeHannum, DNAmAgeSkinBlood, and DNAmTL (Fig. 2d, Supplementary Table S3).

**Fig. 3:**
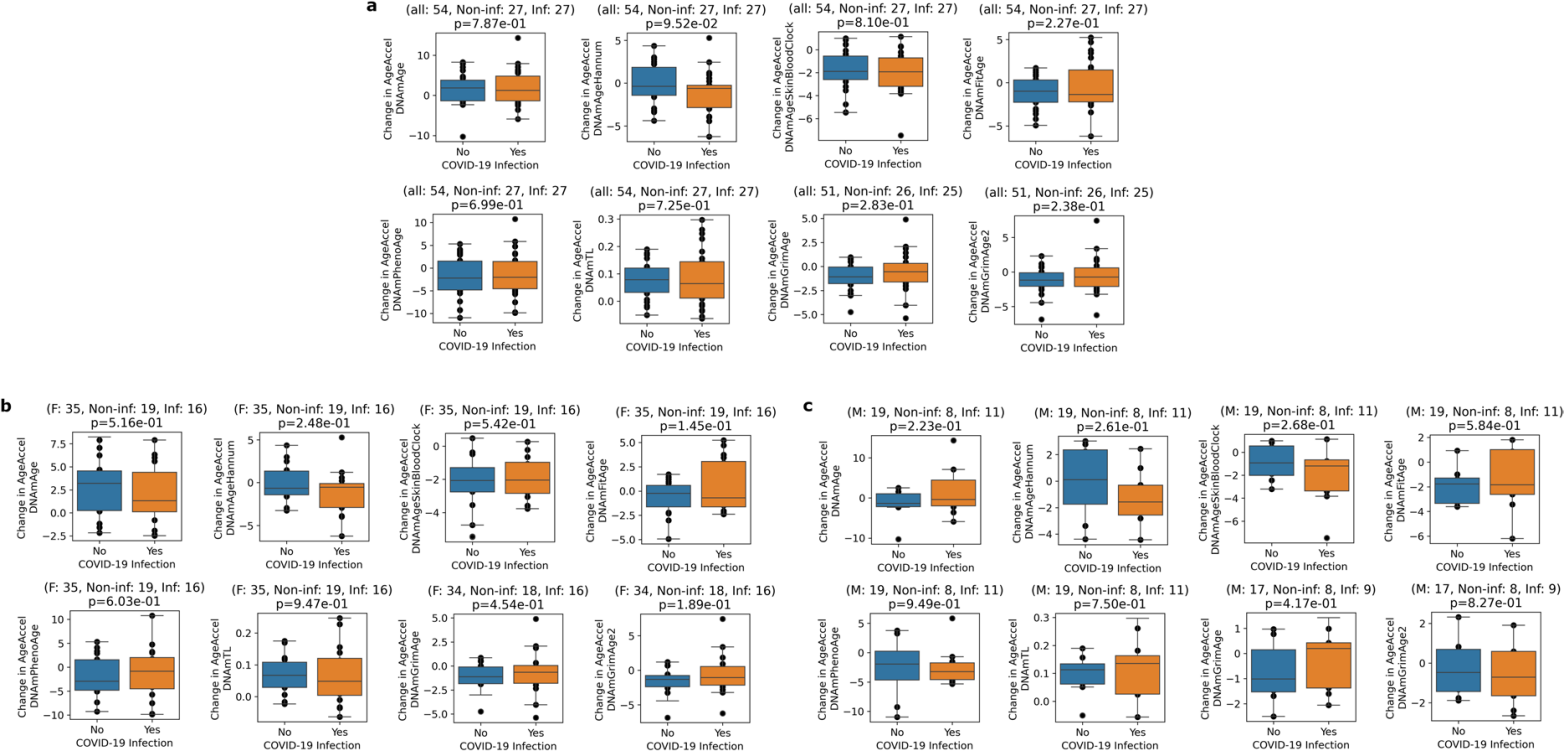
Association of SARS-COV- 2 infection DNA methylation-based aging clock. **a** We directly compared the DNA methylation-based age acceleration changes of SARS-CoV- 2 *infected* and *non-infected* individuals during the examined 3-year-period. We examined eight aging clocks. **b** The same analysis with the separation of genders (*females* and *males*)

To examine the long-term effects of COVID- 19 on aging rates, we directly compared the epigenetic age acceleration changes of infected and non-infected individuals. We did not observe significant change in any cases (Fig. 3a; Supplementary Table S4), even if we separated the analysis by gender (Fig. 3b, c). However, when we adjusted the analysis for baseline variables (age, gender, VO2 max BEFORE, max jump BEFORE, max grip BEFORE, cognition BEFORE, BMI BEFORE, years of regular training, education degree, and high blood pressure) by using multiple linear regression modeling (OLS) of the 3-year change for each prediction value, we found that having COVID- 19 during the 3-year study period significantly increased the change of DNAmGrimAge (*p* = 0.047), DNAmGrimAge2 (*p* = 0.024), and DNAmFitAge (*p* = 0.032). The data suggest that COVID- 19 may have a mild long-term effect on epigenetic aging.

## Discussion

We studied the relationship between physical fitness levels and DNA-methylation-based aging clocks just before the appearance of the COVID- 19 pandemic [10]. We recalled and measured our subjects 3 years after the first sampling and had an opportunity to study the possible effects of COVID- 19 infection on the level of physical fitness and DNA methylation. Surprisingly, the data revealed that male participants or subjects who did not contract COVID- 19 showed an improvement in vertical jump performance—an indicator of lower extremity explosive strength—over the 3-year period, likely due to increased physical activity during the pandemic. This finding was supported by DNA methylation-based aging clocks (DNAmAgeSkinBlood, DNAmFitAge, PhenoAge, and DNAmTL), which also showed slowed aging in males or non-infected subjects. One of the novel findings of the longitudinal investigation of the current study is that COVID- 19 infection significantly increased the speed of aging assessed by DNAmGrimAge, DNAmGrimAge2, and DNAmFitAge compared to non-infected subjects during the 3 years. The long-term effects of COVID- 19 are still not well known, indeed it has been reported that the virus can be identified in some tissues long after acute infection and the reactivation could have serious consequences including enhanced inflammation [11, 12] and accelerated aging assessed by Horvath’s clock [13]. The complexity of the COVID- 19 infection can be judged by the fact that investigations that studied DNA methylation have diverse results [13–15]. Moreover, the long-term consequences of COVID- 19 infections can be attributed to in the regulation of interferon-induced protein 44, hence the orchestration of immune response and can be also involved in the dysregulation of the immune system [15].

When we evaluated the 3-year changes in age acceleration, DNAmAge showed accelerated aging; however, when we separated the analysis by gender, we observed a stronger effect in females with no change in males. This may reflect sex-specific responses to the COVID- 19 pandemic, potentially influenced by differences in stress resilience, and behavioral adaptations during the pandemic. Lifestyle changes (e.g., decreased fitness) may be confound or coupled with SARS-CoV- 2 infection and accelerated epigenetic aging during the pandemic.

When DNA methylation-based aging was examined over the 3-year period, we found a significant decrease in methylation of the promoter region of the H1 FNT gene, which encodes the testis-specific histone H1 family member N (H1 FNT) and plays a crucial role in spermatogenesis [11]. The H1 FNT gene is a potent regulator of cell/tissue-specific gene expression, especially in gastrointestinal organs [16]. Interestingly, our recent study on rats showed that exercise increases protein kinase B and endothelial nitric oxide synthase protein levels and the number of caveolae in the smooth muscles of the intestine [17]. However, further information is needed to explain how H1 FNT is related to the aging process.

We also observed that the promoter methylation of CSTL1 gene increased during the 3-year period. The promoter of CSTL1, which encodes Cystatin-like 1, showed the most significant increase. In humans, this protein is a member of the cystatin “superfamily,” which encompasses proteins that contain multiple Cystatin-like sequences and are generally active cysteine protease inhibitors [18]. This superfamily is associated to coagulation [19], and it was reported that protein content of the extracellular vesicles is also associated with coagulation [20]. However, the possible involvement of the CSTL1 gene in the aging process is yet to be studied.

In summary, this 3-year study shows that infection with COVID- 19 accelerates the DNA methylation-based aging rates. This observation further suggests environmental factors, such as infections by COVID- 19 create long-term effects on DNA methylation which could alter the adaptive immune response for upcoming diseases or expression of prominent genes associated with aging. It is possible that epigenetic clocks can monitor post-COVID recovery at least regarding age acceleration. Finally, we observed that, increased physical activity during the pandemic increased the performance of some physiological functions and attenuated the aging process of male subjects.

## Methods

The study was approved by the National Public Health Center in accordance with the Helsinki Declaration and the regulations applicable in Hungary (25167–6/2019/EÜIG). A total of 54 participants (35 females and 19 males) were recruited for this study. The requirement was done by recalling the Hungarian (control) participants of our previous study [21]. The average age of participants at the time of the first measurement was 58.8 years for all, 61.2 years for females, and 54.3 years for males. At the second measurement (3 years later), the average age was 61.7 for all, 64.1 years for females, and 57.3 years for males. Participants were categorized into two groups based on SARS-CoV- 2 infection status. COVID- 19 group (*infected*): 27 participants who experienced COVID- 19 during the 3-year study period; Control group: 27 participants who did not experience COVID- 19 during the 3-year study period (*non-infected*).

### Physiology tests

The digit span test was applied to assess the working memory, where larger values indicate better memory. Maximum hand gripping force is often used to measure age-associated declines in general muscle strength. The dynamic strength of the legs was assessed by the maximum vertical jump, using a linear encoder. Body mass index was appraised by body composition monitor BF214 (Omron, Japan). Maximal oxygen uptake, VO_2_max, measures the volume of oxygen the body processes during incremental exercise in milliliters used in 1 min of exercise, per kilogram of body weight (mL/kg/min) and was estimated through the Chester step test. Participants were classified into fitness groups by already established sex and age strata-specific VO_2_max reference standard, as described earlier [10].

### Measurements of DNA methylation

The DNA isolation and methylation measurements of the blood samples shipped by us were done by the Genomics Core Facility of Erasmus MC, University Medical Center Rotterdam, NL. The methylation measurements were performed using the Illumina Infinium MethylationEPIC (850 K) BeadChip according to the manufacturer’s protocol, as described previously [10]. Here, we used 54 methylomes from our previous methylation study referred to as MET2019 [10]. The 3-year follow-up samples of the same individuals were measured in another batch (referred to as MET2022) with the same method.

### Epigenetic biomarkers

Epigenetic clocks were applied using the DNA methylation age calculator of the Clock Foundation Team (https://dnamage.clockfoundation.org/clock). We applied the Horvath pan-tissue clock [22], the blood-based Hannum clock [23], the skin and blood clock [24], the PhenoAge clock [25], the DNAmFitAge clock [26], and the GrimAge v1 and v2 clocks [2, 3]. We calculated age acceleration as the residual, per sample, after fitting the predicted age to chronological age (i.e., the age acceleration is the deviation from the trend). The telomere length was also evaluated by using the DNA methylation age calculator, which estimated the telomere length from methylation data [27].

### Normalized beta values

Raw methylation signal intensities were retrieved using the function read.metharray.exp of the *minfi* v1.40.0 *R* package, followed by linear dye bias correction and noob background correction to account for technical variation in background fluorescence signal (Aryee et al. 2014). Specifically, the *β*-value was calculated from the intensity of the methylated and unmethylated sites, as the ratio of fluorescent signals.

### Differently methylated promoter analysis

For the differently methylated promoter analysis, we merged the normalized beta values of the batches MET2019 and MET2022. We considered a CpG site to be located in a promoter region if it was annotated as TSS1500, TSS200, 5′UTR, or 1 stExon [28]. We used FDR correction for the *p*-values.

### Statistical analysis

Python packages were used for statistical analysis. We used two-sided independent Student’s *t*-tests to compare two groups. We used paired *t*-test when “*Before COVID*” and “COVID” samples were compared to each other. We also indicated the matched samples of the same individuals by dashed lines. If *p* values were indicated by an asterisk, we used the notations as follows: ns, *p* > 0.05; *, 0.01 < *p* ≤ 0.05; **, 0.001 < *p* ≤ 0.01; ***, *p* ≤ 0.001, ****, *p* ≤ 0.0001. We did not correct *p*-values for multiple-test comparisons, except for the differently methylated CpG site analysis (FDR).

## Supplementary Information

Below is the link to the electronic supplementary material.

Supplementary Figure S1 Principal component analysis (PCA) of the 54 methylomes of the study. Gender (M – males, F - females) and the time point of the measurement (Before COVID, and COVID) are indicated (CSV 1520 KB)

Supplementary Figure S2. Comparison of mean methylation levels of gene promoters just before COVID- 19 („Before COVID”) and three years later („COVID”) (CSV 1141 KB)

Supplementary Figure S3. Comparison of mean methylation levels of gene promoters just before COVID- 19 („Before COVID”) and three years later („COVID”), only the participants are considered who had COVID- 19 (i.e., infected) (CSV 1161 KB)

Supplementary Figure S4. Comparison of mean methylation levels of gene promoters just before COVID- 19 („Before COVID”) and three years later („COVID”), only the participants are considered who had no COVID- 19 (i.e., non-infected) (CSV 2667 KB)

Supplementary Figure S5. Predictions of the seven epigenetic aging clocks (DNAmAge, DNAmAgeHannum, DNAmAgeSkinBloodClock, DNAmFitAge, DNAmPhenoAge, DNAmGrimAge, DNAmGrimAge2) and the DNA methylation-based predictor of telomere length (DNAmTL). The prediction performance was measured by the Pearson correlation coefficient (r) and mean absolute error (MAE). We calculated age acceleration as the residual, per sample, after fitting the predicted age to chronological age (i.e., the age acceleration is the deviation from the trend) (CSV 17965 KB)

Supplementary file1 (PDF 1.29 MB)

## Data Availability

Data used for the analysis is available in Supplementary Tables S1–S5. Raw and processed methylation data will be available on the GEO database upon publication (GSE293007).
