# Supplementary material for: Associations of epigenetic aging and COVID- 19: A 3-year longitudinal study
Source: GeroScience. 2025 Apr 10;47(3):4889–98. doi: 10.1007/s11357-025-01635-4 (PMC12181606; doi:10.1007/s11357-025-01635-4)
Supplement: Supplementary file 6 — (PDF 1.29 MB) [file 11357_2025_1635_MOESM6_ESM.pdf]

SUPPLEMENTARY FIGURES

OF

Associations of epigenetic aging and COVID-19: a 3-year longitudinal study

Gabor Farkas[1,#], Zahira El Mahdaouy[2,#], Gergely Babszky[1], Matyas Jokai[1], Ferenc Torma[1], Yaodong Gu[3], Ricardo Pinho[4], Ildiko Miklossy[5], Juozas Gordevicius[8], András Benczúr[2], Csaba Kerepesi[2], Zsolt Radak[1,5,6,7,*]

[1]Research Institute of Sport Science, Hungarian University of Sport Science, Budapest, Hungary.

[2]Institute for Computer Science and Control (SZTAKI), Hungarian Research Network (HUN-REN), Budapest, Hungary.

[3]Faculty of Sport Science, Ningbo University, Ningbo, 315211, China.

[4]Laboratório de Bioquímica do Exercício em Saúde, Programa de Pós-Graduação em Ciências da Saúde, Escola de Medicina e Ciências da Vida, Pontifícia Universidade Católica do Paraná, Curitiba, PR, Brasil.

[5]Department of Bioengineering, Sapientia Hungarian University of Transylvania, Piata 26 Libertatii, 530104, Miercurea Ciuc, Romania

[6]Faculty of Sport Sciences, Waseda University, Tokorozawa, Japan.

[7]Institute of Sport Sciences and Physical Education, Faculty of Sciences, University of Pécs, 247624 Pécs, Hungary

[8]Epigenetic Clock Development Foundation, Torrance, California, USA

[#] Joint first authors

*Corresponence: Zsolt Radák, radak.zsolt@tf.hu

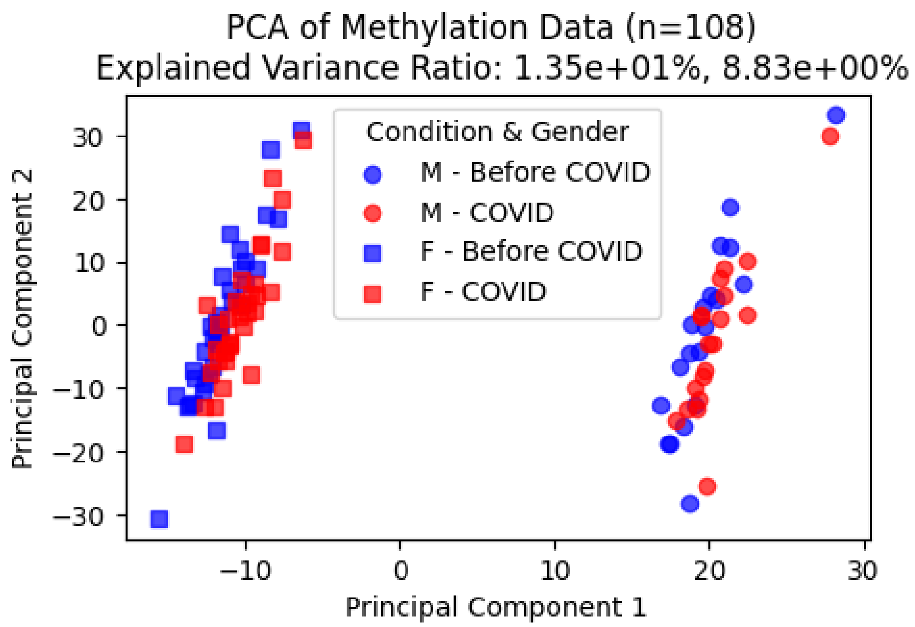

Supplementary Figure S1. Principal component analysis (PCA) of the 54 methylomes of the study. Gender (M – males, F - females) and the time point of the measurement (Before COVID, and COVID) are indicated.

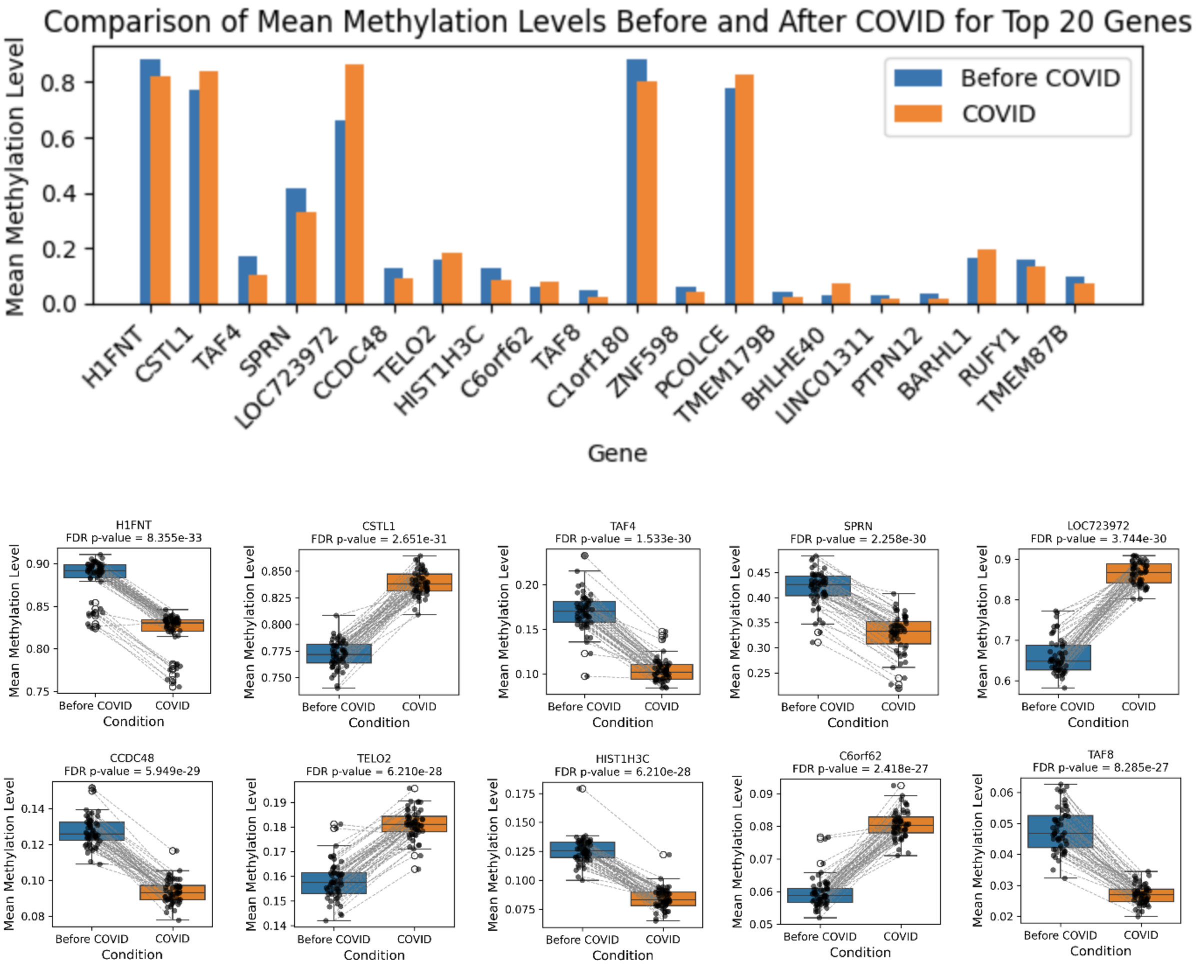

Supplementary Figure S2. Comparison of mean methylation levels of gene promoters just before COVID-19 („Before COVID") and three years later („COVID")

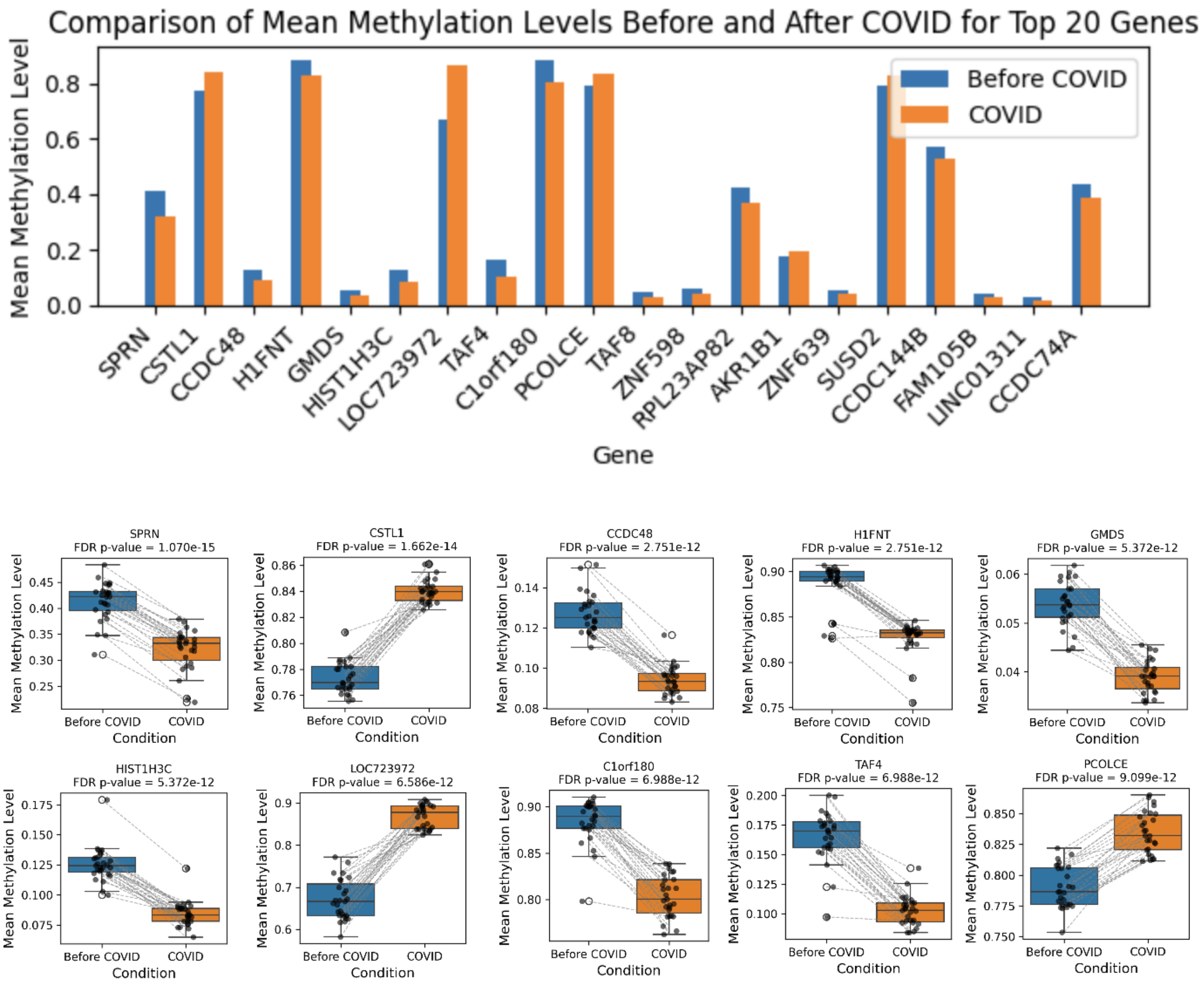

Supplementary Figure S3. Comparison of mean methylation levels of gene promoters just before COVID-19 („Before COVID") and three years later („COVID"), only the participants are considered who had COVID-19 (i.e., infected).

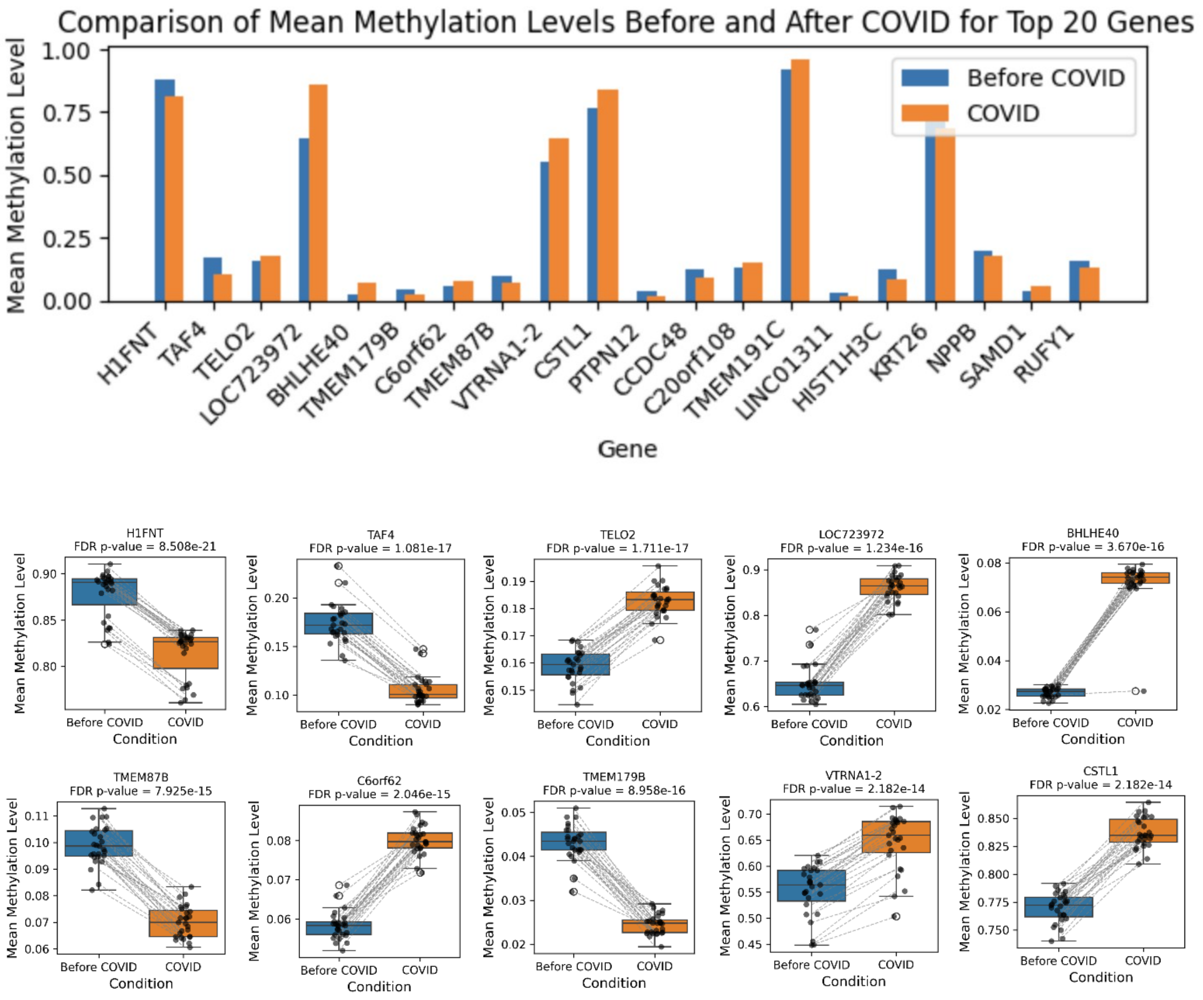

Supplementary Figure S4. Comparison of mean methylation levels of gene promoters just before COVID-19 („Before COVID") and three years later („COVID"), only the participants are considered who had no COVID-19 (i.e., non-infected).

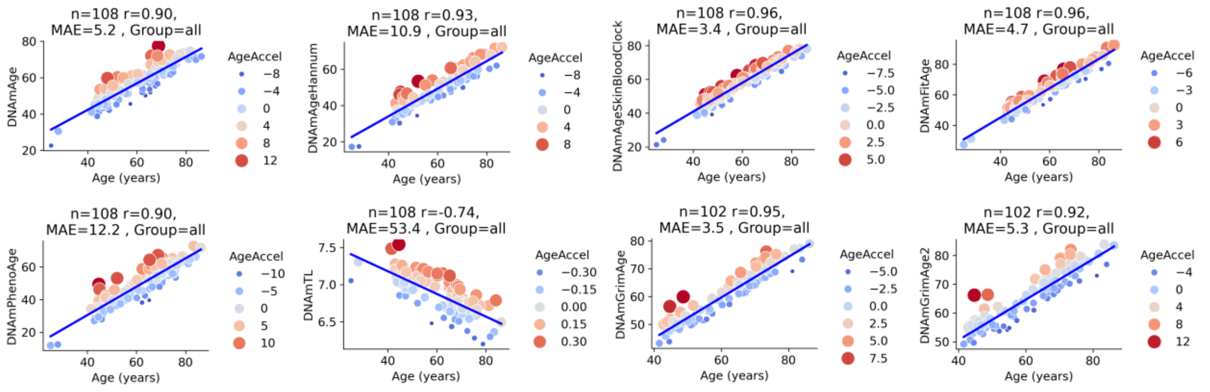

Supplementary Figure S5. Predictions of the seven epigenetic aging clocks (DNAmAge, DNAmAgeHannum, DNAmAgeSkinBloodClock, DNAmFitAge, DNAmPhenoAge, DNAmGrimAge, DNAmGrimAge2) and the DNA methylation-based predictor of telomere length (DNAmTL). The prediction performance was measured by the Pearson correlation coefficient (r) and mean absolute error (MAE). We calculated age acceleration as the residual, per sample, after fitting the predicted age to chronological age (i.e., the age acceleration is the deviation from the trend).
